# Genomic and clinical insights into multidrug-resistant *Corynebacterium striatum* in a psychiatric hospital: development of an exploratory infection probability score

**DOI:** 10.3389/fmicb.2026.1790453

**Published:** 2026-06-15

**Authors:** Guolin Song, Yuting Sun, Fei Yan, Qin Tang, Huarong Yu, Lu Lin, Li Wei, Juan Fang

**Affiliations:** 1 Department of Clinical Laboratory, The Fourth People’s Hospital of Chengdu, Chengdu, China; 2 MOE Key Lab for Neuroinformation, Department of Clinical Laboratory, The Clinical Hospital of Chengdu Brain Science Institute, University of Electronic Science and Technology of China, Chengdu, China; 3 Department of Clinical Laboratory, Chongzhou People’s Hospital, Chengdu, China; 4 Department of Clinical Laboratory, Autobio Diagnostics Co., Ltd., Zhengzhou, China

**Keywords:** colonization versus infection, *Corynebacterium striatum*, infection probability score (IPS), multidrug resistance (MDR), psychiatric hospital, whole-genome sequencing (WGS)

## Abstract

**Background:**

*Corynebacterium striatum* is increasingly recognized as a multidrug-resistant (MDR) opportunistic pathogen, yet its role in psychiatric hospitals remains poorly characterized. Differentiating colonization from true infection in this setting is clinically challenging.

**Methods:**

Thirty *C. striatum* isolates were obtained from psychiatric inpatients with prolonged hospitalization. Antimicrobial susceptibility testing and whole-genome sequencing (WGS) were performed to characterize resistance determinants, virulence-associated genes, and phylogenetic relatedness. Ward distribution was mapped, and core-genome SNP-based phylogenetic analysis was performed. An exploratory Infection Probability Score (IPS) was developed using five routinely available variables, and its apparent performance in the derivation cohort was assessed against a predefined composite clinical reference standard using ROC analysis.

**Results:**

Most isolates displayed MDR phenotypes. Genomic analysis showed concurrent detection of multiple resistance genes together with several putative virulence-associated determinants, including iron-uptake- and adhesion-related genes. Core-genome SNP-based phylogenetic analysis demonstrated genetic diversity across isolates, with incomplete concordance between phylogenetic clustering and ward origin. In exploratory ROC analysis, the composite IPS showed higher apparent discriminatory performance than any single component marker (AUC = 0.795, 95% CI: 0.635–0.955).

**Conclusion:**

This single-center exploratory study provides an initial genomic and clinical characterization of MDR *C. striatum* in long-term psychiatric inpatients. The IPS may offer a clinically interpretable framework for distinguishing infection from colonization in this underexplored setting, but its performance requires confirmation in larger, prospectively designed multicenter studies.

## 1 Introduction

*C. striatum*, a colonizer of the upper respiratory tract and skin conventionally considered an opportunistic pathogen, has recently demonstrated a global trend shifting from low virulence and low clinical attention to a multidrug-resistant (MDR) pathogen associated with a high disease burden in healthcare settings (Milosavljevic et al., 2021; Orosz et al., 2022; Silva-Santana et al., 2021). While previous evidence has been derived primarily from intensive care units (ICU), elderly populations, and oncology patients (Milosavljevic et al., 2021; Silva-Santana et al., 2021), the large and distinct population within specialized psychiatric hospitals, characterized by long-term institutionalization, has been largely overlooked. The infection ecology in psychiatric inpatients is notably vulnerable: prolonged sedation (often via antipsychotics or benzodiazepines) and polypharmacy have been linked to depressed airway protective reflexes and increased aspiration pneumonia risk, while comorbid cognitive and swallowing impairments in this group further elevate vulnerability (Rajamäki et al., 2020). This vulnerability is compounded by reduced immune redundancy from chronic low-grade inflammation and metabolic comorbidities. Within closed wards, high-density living conditions and recurrent antimicrobial exposure create a vicious cycle of high colonization pressure, cross-transmission, and selective pressure for resistance. These factors converge to create a confined micro-ecology characterized by high rates of colonization, exposure, and resistance, which is distinctly different from that of general hospital wards (Rovers et al., 2020; Schinas et al., 2023). In this specific setting, the absence of systematic characterization of the antimicrobial susceptibility profile and virulence gene repertoire of *C. striatum*, together with the lack of reliable clinical tools to discriminate colonization from infection, contributes to concurrent overtreatment and undertreatment. Against this background, our study was designed to provide an initial characterization of MDR *C. striatum* in long-term psychiatric inpatients by integrating phenotypic susceptibility testing, whole-genome sequencing, and routinely available clinical biomarkers. Specifically, we aimed (1) to describe the antimicrobial resistance profile, virulence-associated gene repertoire, and genetic relatedness of *C. striatum* isolates in this underexplored hospital setting, and (2) to develop an exploratory, clinically interpretable Infection Probability Score (IPS) for preliminary discrimination between colonization and infection using routinely available inflammatory markers together with semi-quantitative bacterial load. Rather than proposing a definitive diagnostic instrument, this study was intended as a single-center exploratory analysis to generate hypotheses and inform future validation in larger cohorts.

## 2 Materials and methods

### 2.1 Study design and setting

This study analyzed thirty clinical isolates of *C. striatum* collected from the lower respiratory tracts of long-term inpatients at a tertiary psychiatric hospital in China. All isolates were collected over a 4-month period between March and June 2025. The source patients were housed in closed wards with hospitalization durations ranging from 15 to 759 days (mean, 369.9 days), reflecting prolonged exposure in this cohort. To minimize bias from saliva or upper respiratory tract contamination, all specimens underwent microscopic quality control and were required to have a white blood cell to epithelial cell ratio > 2.5 (Schinas et al., 2023).

### 2.2 Phenotypic analysis

Antimicrobial susceptibility was determined by the broth microdilution method. Minimum inhibitory concentrations (MICs) were interpreted according to the Clinical and Laboratory Standards Institute (CLSI) M100 guidelines. Infection markers—including peripheral white blood cell count (WBC), neutrophil percentage (NEU%), serum procalcitonin (PCT), and C-reactive protein (CRP) (Vassallo et al., 2021)—were quantified in adherence with internal and internationally recognized quality control standards.

### 2.3 Development of the infection probability score

The Infection Probability Score (IPS) was developed as an exploratory and clinically interpretable score for long-term psychiatric inpatients. Five prespecified variables were included on the basis of routine clinical availability and clinical relevance: procalcitonin (PCT), C-reactive protein (CRP), white blood cell count (WBC), neutrophil percentage (NEU%), and semi-quantitative bacterial load.

All biomarker measurements were obtained from routine blood tests performed within 24 h of respiratory specimen collection ( ± 12 h). Microbiological culture results and bacterial-load assessment were derived from the same qualified respiratory specimen collected during this interval.

Bacterial load was assessed semi-quantitatively on blood agar plates using specimen-specific colony-forming unit (CFU) thresholds. For qualified sputum samples, > 10^7^ CFU/mL was considered a high bacterial burden; for bronchoalveolar lavage fluid (BALF), the corresponding threshold was > 10^4^ CFU/mL. These thresholds were adapted from specimen-type-based respiratory microbiology conventions rather than from *C. striatum*-specific validation studies. Values below these thresholds were considered more consistent with colonization (Peng et al., 2020).

Case classification was based on a predefined composite clinical reference standard (Mandell et al., 2007). A case was classified as infection only when all of the following criteria were met: (1) a documented clinical diagnosis of pneumonia or lower respiratory tract infection; (2) radiographic evidence of pulmonary inflammation or consolidation; and (3) isolation of *C. striatum* as the sole pathogen from a qualified respiratory specimen. The requirement for mono-isolation of *C. striatum* was used as a conservative microbiological criterion in this exploratory study to improve specificity of case labeling; however, mono-isolation from respiratory specimens should not be interpreted as definitive proof of causality. Cases not meeting all three criteria were classified as colonization. To reduce microbiological confounding, cases with co-detection of other potential respiratory pathogens in the index respiratory specimen were excluded from IPS analysis. This restrictive framework was chosen to maximize specificity of case labeling in a small exploratory derivation cohort, but it may also have narrowed the clinical spectrum represented in the final analytic dataset. The final analytic cohort consisted of 17 infection cases and 13 colonization cases.

The candidate IPS biomarkers were not explicit components of the formal reference rule. However, because the study was retrospective and case classification was based on routine clinical records rather than blinded adjudication, complete independence between candidate predictors and the final clinical classification could not be guaranteed.

Because of the limited sample size and the exploratory purpose of the study, each variable was dichotomized at its ROC-derived optimal cut-off and assigned 1 point. This equal-weight structure was chosen to preserve interpretability and limit model complexity in a small derivation cohort. We acknowledge that this simplified scoring strategy sacrifices part of the information contained in the original continuous variables and should therefore be regarded as a pragmatic exploratory approach rather than an optimized statistical model. The final IPS therefore ranged from 0 to 5.

### 2.4 Genomic analysis

Whole-genome sequencing (WGS) was performed on all isolates included in the genomic analysis. Genomic DNA was extracted using a magnetic bead-based nucleic acid extraction kit (MD049, Jiyi Biotechnology, China), quantified using the Qubit X-Green II dsDNA Quantitation Kit, and used for preparation of 300–500 bp PCR-free libraries. Sequencing was carried out on the NovaSeq 6000 platform using a paired-end 150-bp strategy (PE150). Raw sequencing reads were quality-filtered using fastp (v0.20.1), including adapter trimming, removal of low-quality reads, trimming of read ends with mean Q scores < 30, and exclusion of reads with post-trimming lengths < 70% of the original read length. *De novo* genome assembly was performed using SPAdes (v3.13.0), and assembly quality was assessed using QUAST (v5.0.2) and BUSCO (v4.1.2). One submitted sample was excluded from downstream genomic analysis because species-purity assessment suggested mixed genomic content. Genome annotation was performed using Prokka (v1.14.6). Putative antimicrobial resistance genes and virulence-associated genes were identified by aligning predicted protein sequences against the CARD and VFDB databases using DIAMOND. For molecular epidemiological analysis, pan-genome analysis was performed using the Roary pipeline to identify the conserved gene set shared across isolates. Core-genome regions were then determined by aligning assembled genomes to the reference/core-gene set using the NUCmer component of MUMmer (v3.1). SNPs within the core genome were extracted, and evolutionary distances among isolates were estimated using FastTree (v2.1.11) under the generalized time-reversible (GTR) model. An approximately maximum-likelihood phylogenetic tree was subsequently constructed and annotated by ward of origin to assess genetic relatedness among isolates and to explore possible intra-hospital dissemination patterns. No contig-level linkage analysis, plasmid characterization, operon-level analysis, or prespecified phylogenetic threshold for cluster definition was performed; therefore, the genomic findings were interpreted descriptively.

### 2.5 Statistical analysis

All statistical analyses were performed using R software (version 4.2.2; R Foundation for Statistical Computing, Vienna, Austria). Receiver operating characteristic (ROC) curves and areas under the curve (AUCs) were generated using the pROC package. Optimal cut-off values were determined using the Youden index. Sensitivity, specificity, and their corresponding 95% confidence intervals were calculated where applicable. The IPS was assessed as an exploratory score in the derivation cohort, and its performance was summarized using ROC analysis, AUC, sensitivity, specificity, prevalence, and a confusion matrix. Because of the limited sample size, formal ROC-curve comparison, calibration assessment, regression-based coefficient estimation, optimism-corrected AUC analysis, and decision curve analysis were not performed. Data visualization was performed using ggplot2 and ComplexHeatmap. Additional supporting information is provided in the Supplementary material.

## 3 Results

### 3.1 Clinical and demographic profile

The cohort comprised 30 long-term psychiatric inpatients with markedly prolonged hospital stays (mean, 369.9 days; range, 15–759). Patients were predominantly older (mean age, 78.7 years; range, 32–94) and mostly diagnosed with organic mental disorders (73.3%), with smaller proportions of schizophrenia and major depressive disorder (each 10.0%). Nearly all patients received long-term sedative medication (96.7%), and comorbidities were common, particularly metabolic disorders (83.3%), cardiovascular disease (83.3%), and chronic respiratory disease (60.0%). Collectively, these features suggest compromised host resilience and weakened respiratory defense, which may predispose this population to colonization and infection with *C. striatum* (Table 1).

**TABLE 1:** Demographic and clinical characteristics of long-term psychiatric inpatients with *C. striatum* isolates (*n* = 30).

Variable	Value
Age (mean, range)	78.67 (32–94)
Sex (male/female)	19 (63.3%) / 11 (36.7%)
Primary psychiatric diagnosis	
Schizophrenia	3 (10%)
Bipolar disorder	1 (3.3%)
Major depressive disorder	3 (10%)
Alzheimer’s disease	23 (76.7%)
Use of long-term sedative medications	29 (96.7%)
Length of hospitalization (days)	Mean: 369.9; Range: 15–759
Comorbid metabolic disorders	25 (83.3%)
Comorbid cardiovascular disease	25 (83.3%)
Comorbid chronic respiratory disease	18 (60.0%)

### 3.2 Antimicrobial resistance patterns

**FIGURE 1:**
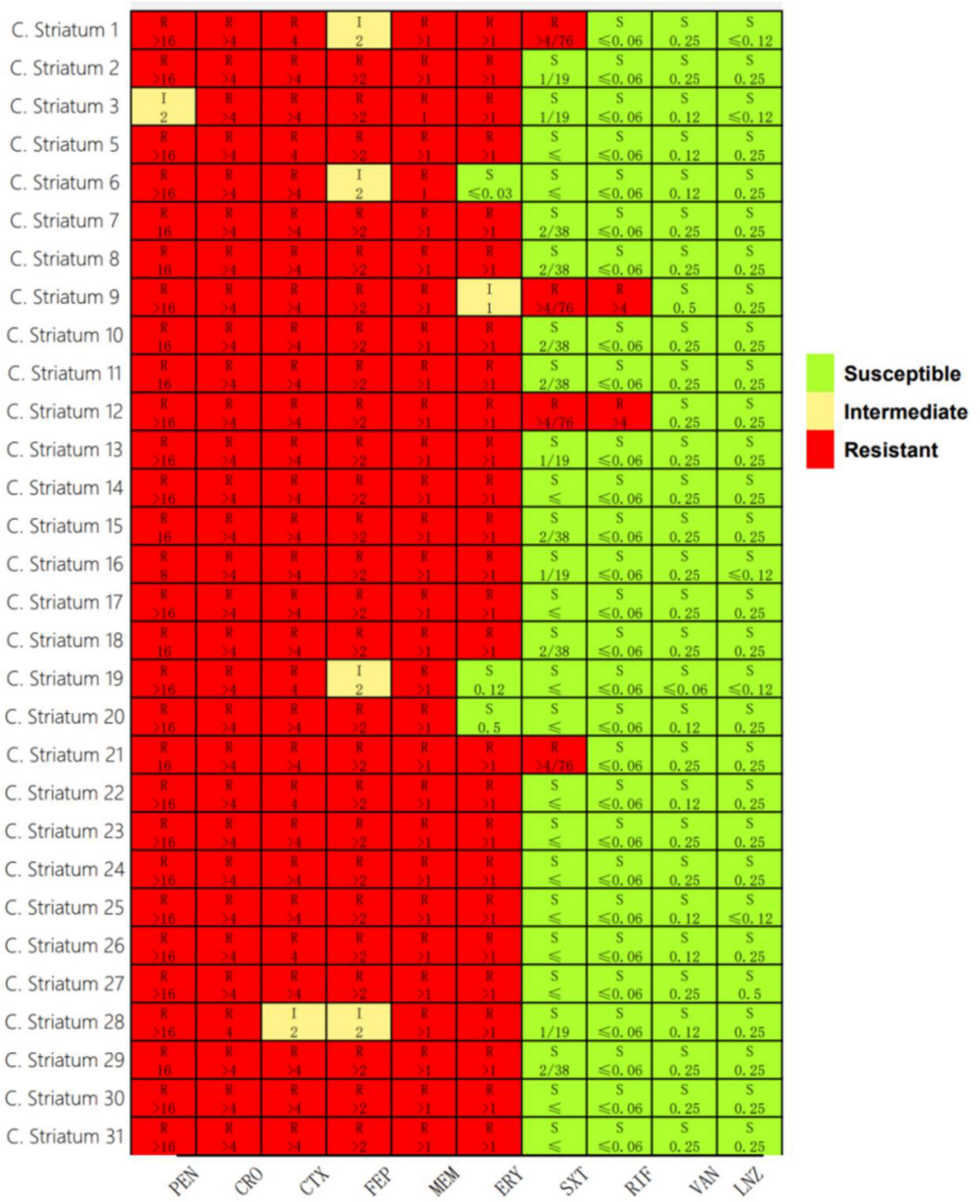
Heatmap of antimicrobial susceptibility patterns among 30 clinical *C. striatum* isolates. The heatmap shows the minimum inhibitory concentration (MIC) values of each isolate against the tested antimicrobial agents. MIC values displayed in each cell are expressed in μg/mL. Colors indicate susceptibility categories according to CLSI interpretive criteria: green, susceptible (S); yellow, intermediate (I); red, resistant (R).

The 30 *C. striatum* isolates demonstrated high rates of resistance to most β-lactams, including penicillin (PEN), ceftriaxone (CRO), cefotaxime (CTX), cefepime (FEP), and meropenem (MEM) (Figure 1). This, combined with near-universal resistance to erythromycin (ERY) (∼100%), established a typical multidrug-resistant (MDR) profile. In contrast, all isolates remained fully susceptible to vancomycin (VAN) and linezolid (LNZ), establishing them as primary therapeutic options. Substantial activity was also retained for rifampin (RIF) and trimethoprim-sulfamethoxazole (SXT). Although a small number of isolates exhibited intermediate susceptibility to certain agents (e.g., CTX, ERY), this proportion was minimal and did not alter the overall MDR classification.

Previous studies have reported that while β-lactam resistance is high among isolates from intensive care units (ICUs), some retain susceptibility to fluoroquinolones or macrolides (Alibi et al., 2017; Asgin and Otlu, 2020). The isolates in our cohort, however, demonstrated nearly universal resistance across these classes. This pattern may reflect the combined effects of prolonged antimicrobial exposure and transmission pressure within a closed hospital environment.

### 3.3 Genomic insights

**FIGURE 2:**
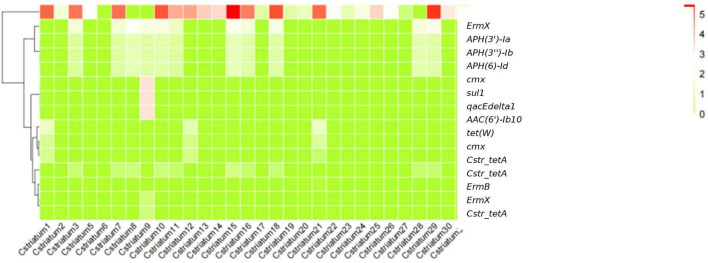
Distribution of antimicrobial resistance genes among 30 clinical *C. striatum* isolates. The heatmap illustrates the distribution patterns of identified antimicrobial resistance genes across the isolates. The dendrogram on the left represents hierarchical clustering of ARGs based on their occurrence patterns among isolates. Each row is labeled with the corresponding gene name.

**FIGURE 3:**
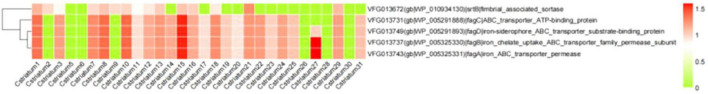
Heatmap of virulence-associated genes among 30 clinical *C. striatum* isolates. The heatmap illustrates the distribution patterns of identified virulence-associated genes across the isolates. The dendrogram on the left represents hierarchical clustering based on gene occurrence patterns. Each row is labeled with the corresponding gene name and predicted function.

Whole-genome sequencing showed that the observed MDR phenotype in the *C. striatum* isolates was accompanied by carriage of multiple antimicrobial resistance genes (Figure 2). Detected macrolide resistance genes included ermX and ermB; aminoglycoside resistance genes included aph(3’)-Ia, aph(3”)-Ib, aph(6)-Id, and aac(6’)-Ib; and tetracycline resistance genes included tet(W) and Cstr_tetA. In addition, the sulfonamide resistance gene sul1 was identified. Together, these findings indicate frequent carriage of multiple resistance determinants across the study isolates. Virulence-associated gene analysis further showed frequent detection of iron-uptake-related genes and adhesion-associated determinants, including the fagABCD cluster and srtB (Figure 3). Because the present analysis was based on gene-detection profiles rather than structural genome analysis, these findings should be interpreted as descriptive co-occurrence rather than evidence of physical linkage between resistance and virulence determinants.

Virulence gene analysis showed that most isolates harbored the iron-uptake-related fagABCD gene cluster and the pilus-associated gene srtB (Figure 3). These virulence factors may enhance bacterial competitiveness in iron-limited environments and promote adherence and colonization on respiratory mucosa.

**FIGURE 4:**
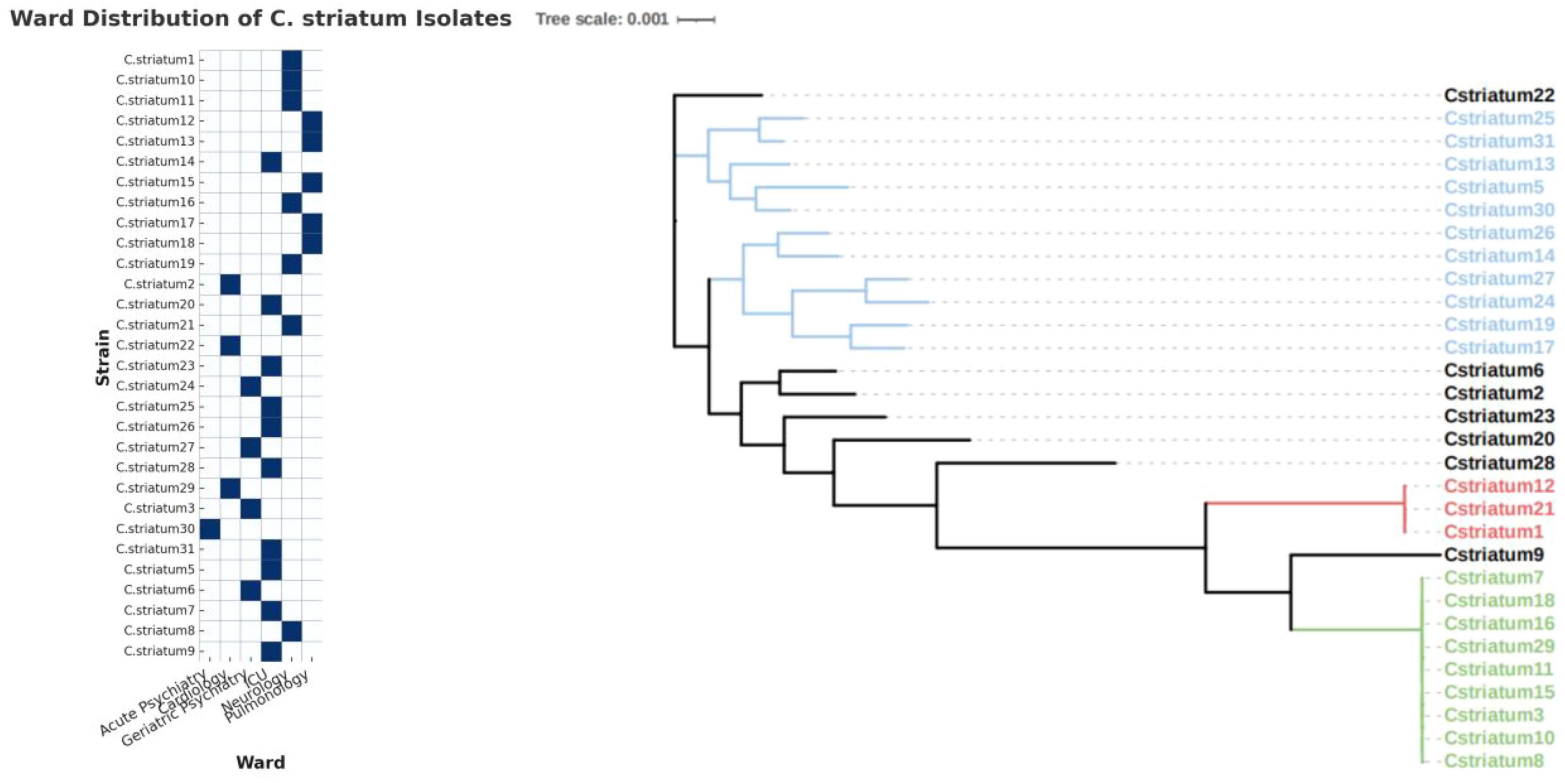
Ward distribution and core-genome SNP-based phylogenetic analysis of 30 clinical *C. striatum* isolates. An approximately maximum-likelihood phylogenetic tree was constructed from core-genome SNPs derived from whole-genome sequencing data to infer genetic relatedness among the isolates. The tree provides a descriptive overview of within-hospital genetic diversity and was not intended to define transmission clusters.

Core-genome SNP-based phylogenetic analysis separated the 30 *C. striatum* isolates into multiple genetic branches, and the clustering pattern showed incomplete concordance with ward origin (Figure 4). Some isolates from the same ward were distributed across different branches, whereas some isolates from different wards were located within the same branch. These findings provide a descriptive overview of genetic diversity within the hospital and are compatible with polyclonal circulation and possible inter-ward dissemination. However, because no formal SNP threshold, cluster definition, temporal cluster reconstruction, or outbreak reconstruction analysis was incorporated, these data should not be interpreted as direct evidence of transmission events or micro-epidemics.

### 3.4 Performance of the infection probability score (IPS)

Based on the 30 patients included in this exploratory study, an Infection Probability Score (IPS) was developed and evaluated using ROC analysis against the predefined composite clinical reference standard. The final analytic cohort included 17 infection cases and 13 colonization cases, corresponding to an infection prevalence of 56.7% in the derivation cohort. The following performance estimates reflect apparent discrimination in the derivation cohort and should therefore be interpreted as exploratory.

**FIGURE 5:**
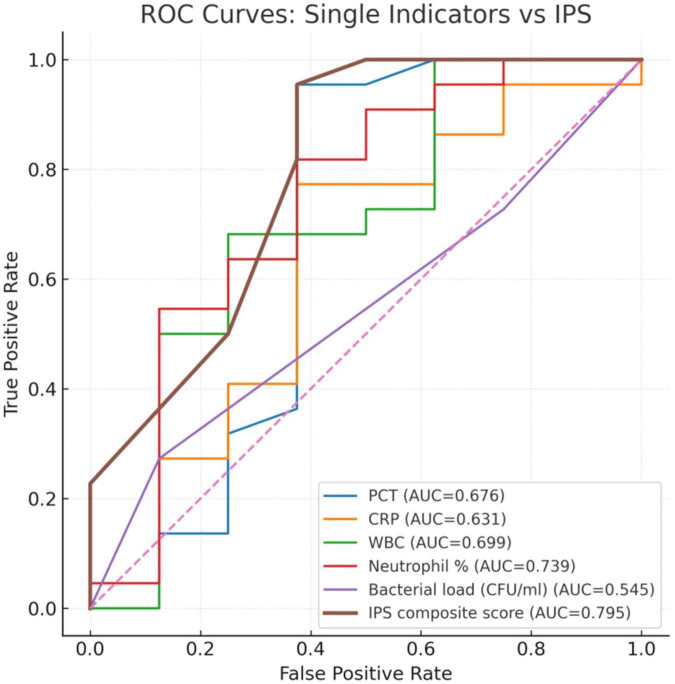
Receiver operating characteristic (ROC) curves for the exploratory Infection Probability Score (IPS) and its component biomarkers. The diagnostic performance of procalcitonin (PCT), C-reactive protein (CRP), white blood cell count (WBC), neutrophil percentage (NEU%), and bacterial load is shown together with the composite IPS. AUC values are presented for each marker and for the composite score. The dashed diagonal line represents no discrimination (AUC = 0.5).

The individual biomarkers showed variable discriminatory performance for distinguishing infection from colonization (Figure 5). The AUC for procalcitonin (PCT) was 0.676 (95% CI: 0.483–0.869), indicating limited discriminatory value. The AUC for C-reactive protein (CRP) was 0.631 (95% CI: 0.430–0.832). White blood cell count (WBC) yielded an AUC of 0.699 (95% CI: 0.511–0.887), while neutrophil percentage (NEU%) showed the highest apparent performance among the individual markers, with an AUC of 0.739 (95% CI: 0.561–0.917). In contrast, bacterial load showed only limited discrimination in this cohort (AUC = 0.545, 95% CI: 0.335–0.755).

When the five variables were combined, the composite IPS showed higher apparent discriminatory performance than any single component marker, with an AUC of 0.795 (95% CI: 0.635–0.955). At the selected cut-off value of ≥ 3, the IPS yielded a sensitivity of 82.4% (95% CI: 56.6–96.2%) and a specificity of 76.9% (95% CI: 46.2–95.0%). The corresponding confusion matrix was as follows: true positives = 14, false positives = 3, false negatives = 3, and true negatives = 10.

Taken together, these findings suggest that, within this small exploratory cohort, combining host inflammatory markers with semi-quantitative bacterial load may provide a more informative framework than reliance on any single indicator alone. However, because the present analysis was performed in a single derivation cohort without formal calibration assessment or external validation, these results should be interpreted as preliminary rather than definitive evidence of clinical utility.

## 4 Discussion

The present study provides an initial descriptive overview of multidrug-resistant *C. striatum* recovered from long-term psychiatric inpatients, including its antimicrobial resistance profile, virulence-associated gene carriage, phylogenetic relatedness, and a preliminary exploratory framework for distinguishing infection from colonization. Within this underexplored setting, the findings are compatible with persistence of *C. striatum* under conditions of host vulnerability and institutional selective pressure. At the same time, given the single-center retrospective design and the limited sample size, the results should be interpreted cautiously and primarily as hypothesis-generating.

From a clinical perspective, the IPS proposed in this study should be viewed as a preliminary and interpretable framework rather than a validated diagnostic tool. In this small single-center derivation cohort, the composite score showed higher apparent discriminatory performance than any individual component marker, suggesting that infection in long-term psychiatric inpatients may not be adequately captured by a single inflammatory marker or by semi-quantitative bacterial burden alone. This is clinically plausible in a population characterized by prolonged hospitalization, chronic sedation, aspiration risk, swallowing dysfunction, and frequent colonization pressure, where signs of infection may be attenuated, delayed, or atypical. Even so, the present estimates remain unstable because they are based on only 30 patients and may be sensitive to small shifts in case classification (Riley and Collins, 2023). The equal-weight structure of the IPS was intentionally adopted to preserve interpretability and to avoid excessive model complexity in a limited derivation dataset, but this simplified approach inevitably sacrifices some of the information contained in continuous predictors. In addition, because the score was evaluated only in the derivation cohort, the reported performance reflects apparent discrimination rather than formal validation. Formal calibration assessment and decision curve analysis were not performed, and therefore the present study does not allow a robust judgment about clinical utility beyond apparent discrimination. Larger, prospectively designed multicenter studies with independent validation cohorts will be needed to determine whether the score retains value in broader clinical settings, to derive more stable predictor weights, and to assess calibration and clinical usefulness more rigorously.

From a pathogen-centered perspective, the genomic analysis showed frequent carriage of multiple resistance genes together with several virulence-associated determinants, including iron-uptake- and adhesion-related genes. These findings may be relevant to persistence and colonization in the respiratory tract, but they should not be overinterpreted (Qiu et al., 2023). Because no contig-level linkage analysis, plasmid characterization, operon-level analysis, or formal co-occurrence statistics were performed, the present data do not demonstrate physical linkage or mechanistic coupling between resistance and virulence determinants. Rather, they provide a descriptive overview of gene carriage patterns in this isolate collection. Similarly, the phylogenetic findings should be interpreted cautiously. The core-genome SNP-based tree provides a descriptive overview of within-hospital genetic diversity rather than formal evidence of transmission dynamics. Although incomplete concordance between phylogenetic clustering and ward origin may be compatible with possible inter-ward dissemination, the current dataset does not allow robust inference of transmission chains, outbreak structure, or micro-epidemics. Future molecular epidemiological studies in this setting would benefit from the integration of temporal sampling, environmental surveillance, and more detailed epidemiological linkage data.

Several limitations should be acknowledged. First, although the reference classification was based on a predefined composite clinical standard requiring documented diagnosis, radiographic evidence, and mono-isolation of *C. striatum*, incorporation bias cannot be excluded (Schiller et al., 2016). The candidate IPS biomarkers were not formal components of the rule itself; however, because classification was retrospective and based on routine clinical records rather than blinded adjudication, inflammatory markers such as WBC, CRP, and PCT may still have influenced the original clinical diagnosis. As a result, the apparent discriminatory performance of the IPS may have been overestimated. Second, the reference framework was intentionally restrictive and may have introduced spectrum bias. By requiring mono-isolation of *C. striatum* and excluding polymicrobial respiratory specimens, the derivation cohort was enriched for more clear-cut cases, potentially under-representing clinically complex infections that are common in real-world respiratory practice. True infections without overt radiographic findings or with co-isolated flora may also have been missed. Because excluded polymicrobial cases were not adjudicated under an alternative reference framework, a robust quantitative sensitivity analysis could not be performed retrospectively. Third, interpretation of *C. striatum* in respiratory specimens remains challenging. As a recognized colonizer of the upper airway and skin, its recovery from lower respiratory tract samples does not by itself establish pathogenicity. In this study, mono-isolation of *C. striatum* was used as a conservative microbiological criterion to improve specificity of case labeling, but this approach cannot fully resolve the distinction between colonization, contamination, and true infection. Likewise, the semi-quantitative CFU thresholds applied to sputum and BALF were adapted from general specimen-type-based respiratory microbiology conventions rather than from species-specific validation studies for *C. striatum* in lower respiratory tract infection (Peng et al., 2020). Contamination and colonization bias may therefore have influenced both microbiological classification and the apparent performance of the IPS. Fourth, to preserve interpretability and reduce model complexity in this small derivation cohort, the IPS used dichotomized predictors with equal weighting. Although practical in an exploratory setting, this structure reduces information carried by continuous variables and may decrease statistical efficiency. Future studies should evaluate broader reference standards, continuous predictor models, repeat isolation, quantitative cultures, specimen quality scoring (Taylor et al., 2025), clinical course response, and expert adjudication in order to define more robust and clinically relevant prediction frameworks.

Taken together, these findings suggest that combining exploratory clinical risk stratification with genomic surveillance may be a useful direction for future work on antimicrobial stewardship and infection prevention in psychiatric hospitals. However, this concept remains preliminary (Rozdilsky et al., 2023). Further prospective studies will be needed to determine how such approaches perform in routine practice and whether they add measurable clinical value in this setting.

## 5 Conclusion

In this single-center exploratory study, *C. striatum* isolates recovered from long-term psychiatric inpatients showed a consolidated multidrug-resistant phenotype together with frequent concurrent detection of multiple resistance genes. By integrating routine inflammatory biomarkers with semi-quantitative bacterial load, we developed an exploratory Infection Probability Score (IPS) that showed higher apparent discriminatory performance than any single component marker in this cohort. However, given the limited sample size, retrospective design, and lack of formal external validation, the IPS should be considered a preliminary, hypothesis-generating framework rather than a validated diagnostic instrument. Future larger, prospectively designed, multicenter studies with independent validation cohorts are needed to confirm the performance of the IPS and clarify its practical value in clinical decision-making.

## Data Availability

The datasets presented in this study can be found in online repositories. The names of the repository/repositories and accession number(s) can be found in the article/Supplementary material.
