# Supplementary material for: Genomic and clinical insights into multidrug-resistant Corynebacterium striatum in a psychiatric hospital: development of an exploratory infection probability score
Source: Front Microbiol. 2026 Jun 15;17:1790453. doi: 10.3389/fmicb.2026.1790453 (PMC13311111; doi:10.3389/fmicb.2026.1790453)
Supplement: Supplementary file 3 [file Table_3.docx]

**细菌全基因组测序结题报告**

**报告出具单位：**杰毅生物技术有限公司

出具日期：2025.06.05

**杭州杰毅生物技术有限公司**

**浙江省杭州市余杭区良渚街道金昌路生命科技小镇2号楼2-4**

一、项目概述

全基因组从头测序(denovo)，不需要任何参考基因组信息即可对某个物种的基因组进行测序，利用生物信息学分析方法进行拼接、组装，获得该物种的基因组序列图谱，并对其进行结构和功能等一系列的分析。本项目对纯培养单菌样本构建300-500bp的PCR-free文库，进行PE150测序。生信分析下机数据质控Q30＞90%，细菌平均1G clean Base,组装Busco完整性评分＞95%，项目交付周期一般25个工作日，后期提供NGS测序技术支持。

二、项目流程


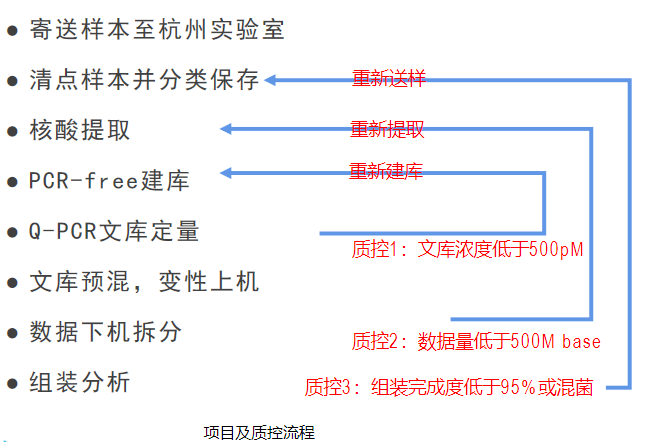


三、测序分析结果

3.1结果汇总

| No | 样本ID | 结果 | 物种 |
| --- | --- | --- | --- |
| 1 | Cstriatum10 | 合格 | Corynebacterium_striatum |
| 2 | Cstriatum11 | 合格 | Corynebacterium_striatum |
| 3 | Cstriatum12 | 合格 | Corynebacterium_striatum |
| 4 | Cstriatum13 | 合格 | Corynebacterium_striatum |
| 5 | Cstriatum14 | 合格 | Corynebacterium_striatum |
| 6 | Cstriatum15 | 合格 | Corynebacterium_striatum |
| 7 | Cstriatum16 | 合格 | Corynebacterium_striatum |
| 8 | Cstriatum17 | 合格 | Corynebacterium_striatum |
| 9 | Cstriatum18 | 合格 | Corynebacterium_striatum |
| 10 | Cstriatum19 | 合格 | Corynebacterium_striatum |
| 11 | Cstriatum1 | 合格 | Corynebacterium_striatum |
| 12 | Cstriatum20 | 合格 | Corynebacterium_striatum |
| 13 | Cstriatum21 | 合格 | Corynebacterium_striatum |
| 14 | Cstriatum22 | 合格 | Corynebacterium_striatum |
| 15 | Cstriatum23 | 合格 | Corynebacterium_striatum |
| 16 | Cstriatum24 | 合格 | Corynebacterium_striatum |
| 17 | Cstriatum25 | 合格 | Corynebacterium_striatum |
| 18 | Cstriatum26 | 合格 | Corynebacterium_striatum |
| 19 | Cstriatum27 | 合格 | Corynebacterium_striatum |
| 20 | Cstriatum28 | 合格 | Corynebacterium_striatum |
| 21 | Cstriatum29 | 合格 | Corynebacterium_striatum |
| 22 | Cstriatum2 | 合格 | Corynebacterium_striatum |
| 23 | Cstriatum30 | 合格 | Corynebacterium_striatum |
| 24 | Cstriatum31 | 合格 | Corynebacterium_striatum |
| 25 | Cstriatum3 | 合格 | Corynebacterium_striatum |
| 26 | Cstriatum4 | 过滤 | Corynebacterium_striatum;Enterococcus_faecium |
| 27 | Cstriatum5 | 合格 | Corynebacterium_striatum |
| 28 | Cstriatum6 | 合格 | Corynebacterium_striatum |
| 29 | Cstriatum7 | 合格 | Corynebacterium_striatum |
| 30 | Cstriatum8 | 合格 | Corynebacterium_striatum |
| 31 | Cstriatum9 | 合格 | Corynebacterium_striatum |
| 32 | PR | 合格 | Staphylococcus_aureus |

注：26怀疑核酸纯度不足，导致测序结果不好。

合格：物种纯度、拼接完整性合格，多物种数据库注释结果一致；

待确定：物种纯度和完整性合格但多物种数据库注释结果存在冲突，需要进一步核查物种是否为新种；

过滤：物种纯度、完整性有任一结果不合格的样本；

物种注释结果：metaphlan、motu、LSU三个数据库注释结果的交集，如果差异大于属水平或者有1个以上数据库未获得注释时，结果为空；

3.2 测序质量评价

| No | 样本ID | CleanReads | CleanBase | CleanQ30 | Depth |
| --- | --- | --- | --- | --- | --- |
| 1 | Cstriatum10 | 13749922 | 1408461457 | 0.972708 | 489.06x |
| 2 | Cstriatum11 | 12871980 | 1296486411 | 0.971634 | 444.26x |
| 3 | Cstriatum12 | 14074772 | 1443796810 | 0.970455 | 500.83x |
| 4 | Cstriatum13 | 15278406 | 1559540263 | 0.968753 | 550.45x |
| 5 | Cstriatum14 | 3026988 | 312613850 | 0.971635 | 112.33x |
| 6 | Cstriatum15 | 14569488 | 1462123635 | 0.970689 | 514.02x |
| 7 | Cstriatum16 | 13510156 | 1327088459 | 0.969867 | 461.13x |
| 8 | Cstriatum17 | 12945400 | 1248084029 | 0.970768 | 437.56x |
| 9 | Cstriatum18 | 15189810 | 1530417148 | 0.968564 | 531.50x |
| 10 | Cstriatum19 | 11832968 | 1141631554 | 0.97147 | 409.34x |
| 11 | Cstriatum1 | 12378052 | 1220306504 | 0.967355 | 426.11x |
| 12 | Cstriatum20 | 10910262 | 1130297060 | 0.970116 | 398.01x |
| 13 | Cstriatum21 | 12790874 | 1325590760 | 0.968591 | 448.48x |
| 14 | Cstriatum22 | 13984858 | 1415652479 | 0.970115 | 507.79x |
| 15 | Cstriatum23 | 13424866 | 1415674651 | 0.968544 | 503.81x |
| 16 | Cstriatum24 | 13482632 | 1453243642 | 0.967496 | 514.06x |
| 17 | Cstriatum25 | 4689900 | 516096828 | 0.961457 | 185.40x |
| 18 | Cstriatum26 | 12519564 | 1342051978 | 0.964009 | 481.02x |
| 19 | Cstriatum27 | 11262594 | 1270847876 | 0.961876 | 443.78x |
| 20 | Cstriatum28 | 12465626 | 1343367024 | 0.969854 | 475.82x |
| 21 | Cstriatum29 | 11216898 | 1234047935 | 0.967938 | 428.87x |
| 22 | Cstriatum2 | 10435368 | 1034478918 | 0.968965 | 365.80x |
| 23 | Cstriatum30 | 10590850 | 1094395694 | 0.964049 | 392.54x |
| 24 | Cstriatum31 | 6530280 | 729497699 | 0.966183 | 263.48x |
| 25 | Cstriatum3 | 11565854 | 1168129536 | 0.969408 | 405.53x |
| 26 | Cstriatum4 | 13827032 | 1481855100 | 0.967732 | 262.84x |
| 27 | Cstriatum5 | 10703648 | 1063921354 | 0.968681 | 376.70x |
| 28 | Cstriatum6 | 11876312 | 1219149476 | 0.96493 | 450.04x |
| 29 | Cstriatum7 | 13703354 | 1409798432 | 0.969021 | 492.37x |
| 30 | Cstriatum8 | 11963674 | 1174940467 | 0.967995 | 408.32x |
| 31 | Cstriatum9 | 16716014 | 1693483819 | 0.971914 | 599.69x |
| 32 | PR | 14521570 | 1686551828 | 0.969786 | 624.96x |

CleanReads：质控后的测序reads数目；

CleanBase：质控后的测序碱基总数目；

CleanQ30：质控后的Q30值，Q30代表准确性大于99.9%的碱基占总碱基的比率；

Depth:测序深度，测序碱基总数和基因组大小的比值，为了获得较好的拼接效果推荐深度
大于100x；
3.3组装质量评价

| No | 样本ID | 完整性（Busco） | 拼接长度 | 预估长度 | contigs | N50 |
| --- | --- | --- | --- | --- | --- | --- |
| 1 | Cstriatum10 | 99.2% | 2879917 | 3,062,929 bp | 98 | 91001 |
| 2 | Cstriatum11 | 98.4% | 2918298 | 3,032,480 bp | 164 | 41511 |
| 3 | Cstriatum12 | 99.2% | 2882790 | 2,999,031 bp | 97 | 87867 |
| 4 | Cstriatum13 | 98.4% | 2833219 | 2,983,130 bp | 96 | 94522 |
| 5 | Cstriatum14 | 99.2% | 2783040 | 2,873,614 bp | 97 | 95683 |
| 6 | Cstriatum15 | 99.2% | 2844510 | 3,018,009 bp | 87 | 96340 |
| 7 | Cstriatum16 | 99.2% | 2877904 | 3,027,003 bp | 90 | 95596 |
| 8 | Cstriatum17 | 99.2% | 2852361 | 2,962,231 bp | 82 | 117272 |
| 9 | Cstriatum18 | 99.2% | 2879457 | 3,118,722 bp | 87 | 101035 |
| 10 | Cstriatum19 | 99.2% | 2788955 | 2,947,272 bp | 83 | 84118 |
| 11 | Cstriatum1 | 98.4% | 2863819 | 3,083,567 bp | 103 | 63139 |
| 12 | Cstriatum20 | 99.2% | 2839893 | 2,919,105 bp | 89 | 86806 |
| 13 | Cstriatum21 | 91.9% | 2955711 | 2,982,109 bp | 359 | 16137 |
| 14 | Cstriatum22 | 99.2% | 2787879 | 2,787,226 bp | 76 | 113055 |
| 15 | Cstriatum23 | 99.2% | 2809923 | 2,820,037 bp | 88 | 92612 |
| 16 | Cstriatum24 | 99.2% | 2826995 | 2,820,920 bp | 88 | 124403 |
| 17 | Cstriatum25 | 97.6% | 2783738 | 2,922,244 bp | 118 | 66793 |
| 18 | Cstriatum26 | 99.2% | 2790041 | 2,740,087 bp | 80 | 106892 |
| 19 | Cstriatum27 | 93.5% | 2863701 | 2,701,961 bp | 197 | 50370 |
| 20 | Cstriatum28 | 99.2% | 2823264 | 2,731,170 bp | 97 | 104964 |
| 21 | Cstriatum29 | 99.2% | 2877439 | 2,755,564 bp | 97 | 76716 |
| 22 | Cstriatum2 | 98.4% | 2827954 | 3,034,457 bp | 103 | 64141 |
| 23 | Cstriatum30 | 99.2% | 2788013 | 2,781,997 bp | 108 | 64101 |
| 24 | Cstriatum31 | 99.2% | 2768675 | 2,594,670 bp | 87 | 74919 |
| 25 | Cstriatum3 | 99.2% | 2880467 | 3,117,437 bp | 95 | 99051 |
| 26 | Cstriatum4 | 100.0% | 5637754 | 3,472,956 bp | 325 | 42164 |
| 27 | Cstriatum5 | 98.4% | 2824338 | 3,035,541 bp | 121 | 60596 |
| 28 | Cstriatum6 | 99.2% | 2708983 | 2,987,243 bp | 92 | 65544 |
| 29 | Cstriatum7 | 99.2% | 2863276 | 2,977,263 bp | 83 | 98973 |
| 30 | Cstriatum8 | 99.2% | 2877528 | 3,027,535 bp | 90 | 99051 |
| 31 | Cstriatum9 | 99.2% | 2823926 | 2,926,468 bp | 119 | 103812 |
| 32 | PR | 99.2% | 2698641 | 2,755,545 bp | 30 | 203929 |

完整性（Busco）：基于细菌保守基因的拼接数目估算组装完整性，参考质控标准为95%。部分物种和参考的保守基因差异较大，在测序深度足够的情况下仍然可能低于95%的参考值，可以根据拼接后的总长度和预估长度的比值来估算拼接的完整性；

Contigs：组装得到的contigs碎片数量，contigs为组装单位代表一段连续无间断的序列。

拼接长度：组装后获得的全部contigs长度和；

预估长度：根据reads中序列多样性估算样本的测序信息量大小；

N50：按照长度对contigs进行排序，长度超过组装总长度一半时对应contigs的长度；该值越大越好。当该值低于平均基因长度（细菌参考900~1200）时，提示使用组装序列进行基因预测的效果较差。

3.4注释信息

| No | 样本ID | 预测编码区 | rRNA | tRNA | miscRNA | CARD注释蛋白 |
| --- | --- | --- | --- | --- | --- | --- |
| 1 | Cstriatum10 | 2663 | 3 | 50 | 9 | {{CARDpro}} |
| 2 | Cstriatum11 | 2693 | 7 | 51 | 9 |  |
| 3 | Cstriatum12 | 2665 | 3 | 55 | 7 |  |
| 4 | Cstriatum13 | 2623 | 3 | 49 | 9 |  |
| 5 | Cstriatum14 | 2554 | 2 | 48 | 8 |  |
| 6 | Cstriatum15 | 2631 | 3 | 50 | 9 |  |
| 7 | Cstriatum16 | 2657 | 3 | 50 | 9 |  |
| 8 | Cstriatum17 | 2627 | 3 | 55 | 8 |  |
| 9 | Cstriatum18 | 2655 | 3 | 50 | 9 |  |
| 10 | Cstriatum19 | 2543 | 2 | 55 | 9 |  |
| 11 | Cstriatum1 | 2653 | 4 | 55 | 7 |  |
| 12 | Cstriatum20 | 2612 | 3 | 47 | 9 |  |
| 13 | Cstriatum21 | 2805 | 7 | 56 | 7 |  |
| 14 | Cstriatum22 | 2566 | 3 | 54 | 9 |  |
| 15 | Cstriatum23 | 2571 | 2 | 53 | 8 |  |
| 16 | Cstriatum24 | 2602 | 4 | 51 | 9 |  |
| 17 | Cstriatum25 | 2560 | 3 | 53 | 10 |  |
| 18 | Cstriatum26 | 2597 | 2 | 54 | 8 |  |
| 19 | Cstriatum27 | 2662 | 2 | 50 | 9 |  |
| 20 | Cstriatum28 | 2631 | 3 | 53 | 9 |  |
| 21 | Cstriatum29 | 2665 | 3 | 51 | 9 |  |
| 22 | Cstriatum2 | 2635 | 3 | 48 | 9 |  |
| 23 | Cstriatum30 | 2597 | 5 | 50 | 9 |  |
| 24 | Cstriatum31 | 2546 | 3 | 48 | 9 |  |
| 25 | Cstriatum3 | 2658 | 2 | 50 | 9 |  |
| 26 | Cstriatum4 | 5385 | 8 | 96 | 62 |  |
| 27 | Cstriatum5 | 2631 | 3 | 53 | 9 |  |
| 28 | Cstriatum6 | 2488 | 2 | 48 | 10 |  |
| 29 | Cstriatum7 | 2639 | 3 | 51 | 9 |  |
| 30 | Cstriatum8 | 2661 | 3 | 50 | 9 |  |
| 31 | Cstriatum9 | 2663 | 3 | 48 | 9 |  |
| 32 | PR | 2497 | 3 | 34 | 103 |  |

预测编码区：Prokka流程预测的基因编码区序列数目；

rRNA：Prokka流程预测的核糖体RNA序列数目；

tRNA：Prokka流程预测的转运RNA序列数目；

miscRNA：Prokka流程预测的小RNA序列数目；

CARD注释蛋白:耐药数据库CARD注释到的耐药蛋白数目；

3.5 序列分型

| No | 样本ID | PubMLST方法分类 | PubMLST分型结果 |
| --- | --- | --- | --- |
| 1 | Cstriatum10 | - | - |
| 2 | Cstriatum11 | - | - |
| 3 | Cstriatum12 | - | - |
| 4 | Cstriatum13 | - | - |
| 5 | Cstriatum14 | - | - |
| 6 | Cstriatum15 | - | - |
| 7 | Cstriatum16 | - | - |
| 8 | Cstriatum17 | - | - |
| 9 | Cstriatum18 | - | - |
| 10 | Cstriatum19 | - | - |
| 11 | Cstriatum1 | - | - |
| 12 | Cstriatum20 | - | - |
| 13 | Cstriatum21 | - | - |
| 14 | Cstriatum22 | - | - |
| 15 | Cstriatum23 | - | - |
| 16 | Cstriatum24 | - | - |
| 17 | Cstriatum25 | - | - |
| 18 | Cstriatum26 | - | - |
| 19 | Cstriatum27 | - | - |
| 20 | Cstriatum28 | - | - |
| 21 | Cstriatum29 | - | - |
| 22 | Cstriatum2 | - | - |
| 23 | Cstriatum30 | - | - |
| 24 | Cstriatum31 | - | - |
| 25 | Cstriatum3 | - | - |
| 26 | Cstriatum4 | efaecium | - |
| 27 | Cstriatum5 | - | - |
| 28 | Cstriatum6 | - | - |
| 29 | Cstriatum7 | - | - |
| 30 | Cstriatum8 | - | - |
| 31 | Cstriatum9 | - | - |
| 32 | PR | saureus | - |

PubMLST方法分类：pubMLST数据库记录的方法scheme，每个scheme对应一个物种分型方法学，部分物种可能有多个scheme分类，比如大肠有ecoli 和 ecoli_achtman_4等，结果“-”代表在pubMLST中未匹配到合适的MLST方法学；

PubMLST分型结果：根据pubMLST方法学进行分型鉴定的结果，结果“数字”代表分型为scheme方法学中的某一ST类别，结果“-”代表分型结果不在已知ST类型中，在匹配到明确的scheme时，排除测序拼接的技术因素后，可以推测为新型ST；

四、实验和分析流程介绍

4.1 实验流程：

接收到标本后，核对标本信息无误使用杰毅生物”核酸提取试剂盒（磁珠法）”货号MD049进行核酸提取。使用“Qubit X-Green Ⅱ dsDNA Quantitation Kit”货号Q2038对核酸进行定量；文库构建使用杰毅生物“宏基因组 DNA 建库试剂盒（可逆 末端终止测序法）”货号MD001按照100-200 ng总量，但总体积不超过35 μL的核酸或其稀释液进行文库构建，文库浓度大于500 pM。使用杰毅生物“纯化试剂盒(磁珠法)”货号MD012进行文库纯化回收。构建好的文库使用杰毅生物“文库定量试剂盒”货号MD057对NGS文库进行Q-PCR定量，定量引物：P5：AATGATACGGCGACCACCGA ；P7：CAAGCAGAAGACGGCATACGA

文库变性复性后，测序策略采用NovaSeq 6000 PE150，使用“NovaSeq Xp 2 泳道试剂盒测序。

4.2、数据分析

4.2.1测序数据预处理

使用fastp1 0.20.^1^默认参数进行下机reads的质控；修剪reads中含Illumina标准Truseq接头的序列；过滤含连续低质量碱基的reads；修剪平均Q值低于30的reads末端；修剪后的reads长度如果低于70 %将被丢弃。

4.2.2基因组组装

使用 SPAdes^2^ 3.13.0 组装软件进行组装分析( Assembly Analysis )；选择21、33、55、77 、99Kmer中的最佳组装结果，滤掉500 bp以下以及深度低于10 x的片段，

使用bandge^3^ 0.8.1对组装结果进行可视化，结果文件为sample.png。

使用QUAST^4^ v5.0.2对组装质量进行评价主要参数contigs代表拼接后碎片数量，该值越低代表拼接完成性越好；Mapped(%)代表拼接碎片使用的reads比率，该值过低，代表存在序列污染或者测序深度不够；N50为组装指标表示contigs从大到小排序，达到Total length 50%时的最后一个加上contigs大小，该值越大越好，为了较好的预测基因，该值应大于平均基因长度；使用BUSCO^5^ 4.1.2对组装结果进行完成度预估，参考框架图完成度标准为95%，部分不常见物种可能低于该值，需要根据拼接基因组大小和预估基因组大小的比值进行估算。

使用jellyfish^6^ (version 2.2.10)和GenomeScope^7^ (version 3.2.0) 软件对测序reads中含有的信息总量进行预估，推测基因组大。

4.2.3基因组注释

使用prokka^8^ 1.14.6对组装结果进行基因预测及通用注释，主要结果为prokka_annot文件夹,其中后缀名fna的sample.fna文件为基因组fasta结果文件；后缀名faa的sample.faa文件为蛋白注释的fasta结果文件；后缀名ffn的sample.ffn文件为基因注释的fasta结果文件；后缀名gff的sample.gff文件为基因注释信息的gff结果文件；后缀名copies的sample.copies文件为基因预估拷贝数结果；

使用diamond^9^程序将蛋白序列分别比对 CARD抗性基因数据库^10^和VFDB致病菌毒力因子数据库^11^进行基因功能注释。使用mlst 2.19.0 程序将基因结果比对到PUBMLST数据库（https://pubmlst.org）进行分型注释^12^。

参考文献：

1. Chen, S., Zhou, Y., Chen, Y. & Gu, J. fastp: an ultra-fast all-in-one FASTQ preprocessor. Bioinformatics 34, i884-i890 (2018)
2. Prjibelski, A., Antipov, D., Meleshko, D., Lapidus, A. & Korobeynikov, A. Using SPAdes De Novo Assembler. Curr Protoc Bioinformatics 70, e102 (2020).
3. Wick, R.R., Schultz, M.B., Zobel, J. & Holt, K.E. Bandage: interactive visualization of de novo genome assemblies. *Bioinformatics* **31**, 3350-2 (2015).
4. Gurevich, A., Saveliev, V., Vyahhi, N. & Tesler, G. QUAST: quality assessment tool for genome assemblies. *Bioinformatics* **29**, 1072-5 (2013).
5. Simao, F.A., Waterhouse, R.M., Ioannidis, P., Kriventseva, E.V. & Zdobnov, E.M. BUSCO: assessing genome assembly and annotation completeness with single-copy orthologs. Bioinformatics 31, 3210-2 (2015).
6. Marcais, G. & Kingsford, C. A fast, lock-free approach for efficient parallel counting of occurrences of k-mers. Bioinformatics 27, 764-70 (2011).
7. Vurture, G.W. et al. GenomeScope: fast reference-free genome profiling from short reads. Bioinformatics 33, 2202-2204 (2017).
8. Seemann, T. Prokka: rapid prokaryotic genome annotation. Bioinformatics 30, 2068-9 (2014).
9. Buchfink, B., Xie, C. & Huson, D.H. Fast and sensitive protein alignment using DIAMOND. Nat Methods 12, 59-60 (2015).
10. Jia, B. et al. CARD 2017: expansion and model-centric curation of the comprehensive antibiotic resistance database. Nucleic Acids Res 45, D566-D573 (2017).
11. Liu, B., Zheng, D., Jin, Q., Chen, L. & Yang, J. VFDB 2019: a comparative pathogenomic platform with an interactive web interface. Nucleic Acids Res 47, D687-D692 (2019).
12. Jolley, K.A., Bray, J.E. & Maiden, M.C.J. Open-access bacterial population genomics: BIGSdb software, the PubMLST.org website and their applications. *Wellcome Open Res* **3**, 124 (2018)
